# Effects of Fertilization and Irrigation on Abiotic Stress and Antioxidant Markers in Leaves of Field-Grown *Cucurbita maxima*, *C. moschata*, and *C. pepo*

**DOI:** 10.3390/plants15192971

**Published:** 2026-09-29

**Authors:** Biljana Kiprovski, Milka Brdar-Jokanović

**Affiliations:** Institute of Field and Vegetable Crops, National Institute of the Republic of Serbia, Maksima Gorkog 30, 21000 Novi Sad, Serbia; milka.brdar@ifvcns.ns.ac.rs

**Keywords:** antioxidants, abiotic stress, *Cucurbita* species, drought, osmoprotectants

## Abstract

The main aim of this study was to evaluate the impact of contrasting irrigation and fertilization conditions on the most commonly tested markers of abiotic stresses: lipid peroxidation intensity (LP), proline (Pro), reduced glutathione (GSH) content, and total phenolics (TP) content in leaves of 25 genotypes of three *Cucurbita* species grown in an open field experiment. The experiment included unfertilized and non-irrigated, fertilized and non-irrigated, unfertilized and irrigated, and fertilized and irrigated treatments. Contrary to irrigated treatments, the intensity of LP was markedly higher in samples that were not irrigated (36.1–52.5%). For all *C. moschata* genotypes and most of the *C. maxima* and *C. pepo genotypes*, accumulation of Pro (0.7–3.0 mg/g) was higher in non-irrigated treatments, while GSH values were mostly higher in fertilized plants. The content of TP was higher in plants that were both irrigated and fertilized (8.6–38.4 mg/g GAE), reaching the highest values in *C. pepo* genotypes. The results indicate that irrigation has a greater impact on analyzed biochemical markers than fertilization in the early flowering phase of tested genotypes.

## 1. Introduction

The world production of pumpkins, squash and gourds in the past years (1994–2024) has increased steadily, with an average production area of 2.3 million ha and production of 34.6 million t, according to the FAOSTAT Database [1]. The most cultivated pumpkin species are *Cucurbita maxima* Duch., *C. moschata* Duch., and *C. pepo* L.

Pumpkins, as alternative oilseed plants and multifunctional food, may hold a significant place in sustainable plant production, which is a part of many EU strategies that support agrobiodiversity, sustainability, food security, and a nature-positive economy. Underutilized vegetables, such as pumpkins, are important for addressing future food-production challenges.

Pumpkin is a warm-season crop that performs optimally under favorable environmental conditions. However, abiotic stress factors, particularly drought and lower soil fertility, can substantially reduce fruit quality and yield. As the frequency and intensity of these stresses continue to increase due to climate change, developing effective strategies to improve crop adaptation has become increasingly important [2].

The main challenges in plant production are abiotic and biotic stresses that, in the form of multi-stresses, hinder the development of many crops. Mittler et al. [3] explained the importance of reactive oxygen species (ROS) as signaling molecules that enable rapid cellular responses to diverse stimuli in plants and the sensing of abiotic and biotic stresses. Singlet oxygen (^1^O_2_), superoxide (O_2_·^−^), hydroxyl radical (HO·), hydrogen peroxide (H_2_O_2_), and various forms of organic and inorganic peroxides represent the major forms of ROS, which are involved in the ROS wave that represents the cell-to-cell enhanced ROS production cascade. Along with hydraulic, electrical, calcium, redox, volatile waves, and chemical communication, ROS waves also participate in systemic cell-to-cell signaling in plants [4,5] during the multifactorial stress combination of abiotic and biotic stresses [6,7].

These molecules, which are formed by excitation or reduction in atmospheric oxygen, integrate environmental signals, activate stress-response networks in plants (cell walls, apoplasts, cytosol, nuclei, and organelles), and strengthen the defense mechanisms and resilience.

Under drought stress and other abiotic stresses, various osmoprotectants (which prevent dehydration of tissues by maintaining turgor) are synthesized. They are accompanied by osmotically active molecules, including soluble sugars, sugar alcohols, and amino acids. Additionally, synthesis of antioxidants is activated, including flavonoids and other phenolic compounds, which are potent scavengers of ROS and other reactive radicals. This leads to modulation of antioxidant activities, increased relative water contents, decreased electrolytic leakage and malondialdehyde contents (the product of cell membrane degradation, peroxidation of membrane lipids), and structural adaptation of membranes [5,8].

Underutilized vegetables are often low-input (low or no artificial fertilizers and pesticide application) with high tolerance to harsh climates and rich in important nutrients (vitamins, minerals, and antioxidants) [9]. During the flowering phase, under heat stress and drought, there is a competition for available assimilates and dominance of basal fruits due to the production of plant growth regulators from the developing fruit, which induce abscission of terminal and intermediate flowers, resulting in a lower number of fruits per plant [10,11].

Although plants of the genus *Cucurbita* are relatively tolerant to heat and drought, these stresses impact their development, especially during flowering and fruit development stages [12]. Understanding plant responses to drought, heat, and their combination is crucial for breeding resistant crop varieties [13]. Biochemical traits that are significantly altered under stress conditions may serve as reliable biomarkers for identification and selection of genotypes with enhanced resilience to abiotic stress.

There are few studies on responses to drought, heat, and osmotic stress in pumpkins grown in an open field at the flowering stage. Most of the published papers explored biochemical markers in pumpkin seedlings or in a considerably smaller number of genotypes, and most of the experiments in published papers were conducted in controlled conditions [14,15,16,17,18,19,20,21]. Since the flowering stage represents one of the crucial stages of development of these species, it was important to test whether the lack of application of fertilization or irrigation would add to stress in tested plants at this stage of development.

The main aim of this study was to evaluate the impact of soil fertilization and irrigation, as well as their combination, on the most commonly tested biochemical parameters, including lipid peroxidation (a marker of oxidative stress), total phenolics (as a group of important antioxidants), proline (a marker of drought stress), and reduced glutathione contents (a proposed marker of abiotic stress tolerance) in leaves of 25 genotypes from three *Cucurbita* species (*C. maxima*, *C. moschata*, and *C. pepo*) grown in an open field. We hypothesized that:

(1) The absence of irrigation enhances lipid peroxidation intensity in tested plant leaves;
(2) Contrasting irrigation and fertilization conditions impact changes in the accumulation of tested osmoprotectants and antioxidants in the early flowering phase of development of tested *Cucurbita* species.

The observed differences among genotypes within species could help in the selection of genotypes that would be evaluated in further breeding programs as promising drought-tolerance candidates, with a view to developing low-input *Cucurbita* production systems.

## 2. Results and Discussion

In addition to their phenotypical divergence, it was important to understand whether the genotypes chosen for the given research can further differ in response to stress in one of the more sensitive phases of development, and whether they can be grouped according to shared patterns when exposed to testing conditions.

Intensity of lipid peroxidation was markedly higher in leaves of non-irrigated plants (36.1–52.5%) than in those from irrigated treatments (Figure 1 and Supplementary Table S2). Additionally, *C. maxima* (five genotypes out of nine) and *C. moschata* (three genotypes out of nine) had higher lipid peroxidation intensity in the F+0I treatment (Figure 1a,b), which was the case for one *C. pepo* as well (Figure 1c). When compared among species, the highest values for MDA content in *C. moschata* were up to 85.29 nmol/g fw (fw—fresh weight), which was 1.4- and 1.5-times higher than in *C. maxima* and *C. pepo*, respectively (Figure 1, Supplementary Figure S1 and Supplementary Table S2).

Biareh et al. [14] tested MDA, hydrogen peroxide (H_2_O_2_), and ion leakage and activities of antioxidant enzymes: superoxide dismutase (SOD), glutathione reductase (GR), catalase (CAT), ascorbate peroxidase (APX) and peroxidase (POX) in leaves of *C. pepo* at the flowering stage of development. They reported that a decrease in available soil water dramatically increased damage in cell membranes and ion leakage (MDA content changed from 2 to 2.5 µmol/g dry weight (dw)).

Differences in LP levels among plants may reflect variation in the amount of polyunsaturated fatty acids, differences in antioxidant defense capacity, and exposure to different stress conditions. Although low basal levels of lipid peroxidation products are normally present in plants and may participate in signaling, their concentration and variability can increase markedly when plants are exposed to biotic or abiotic stress [22].

Proline accumulation was different for all three species, ranging from 0.7 to 3.0 mg/g dw. For all *C. moschata* genotypes and most of the *C. maxima* and *C. pepo genotypes*, accumulation of proline was higher in non-irrigated treatments; however, there was an exception in each species where the highest content was recorded in both irrigated and fertilized treatments (Figure 2 and Supplementary Figure S1). The highest content of proline was recorded in the leaves of irrigated and fertilized *C. maxima* genotype Max113 (Figure 2a). The highest variation in the proline content was observed in the 0F+0I treatment for *C. moschata*.

Proline is a multifunctional amino acid involved in osmoprotection and stress signaling [11,23,24]. In contrast, some authors emphasize that excessively high proline levels, in more sensitive genotypes, may indicate stress-induced metabolic imbalance rather than effective tolerance [25]. Under drought stress, proline accumulation and the induction of proline-rich proteins (PRPs, which can contain up to 70% of proline and are localized in the cell wall) synergistically act in osmotic adjustment, reinforce structural integrity, and enhance cellular resilience [26].

A study conducted in controlled conditions with seedlings of winter squash and pumpkin lines and their hybrid genotypes [17] revealed that proline content in leaves varied from 1.37 to 3.33 mg/g in *C. moschata* genotypes, 2.37 and 2.83 mg/g in *C. maxima* genotypes and 1.21–2.94 mg/g in their hybrids. All results were similar to results obtained in our study (0.74–2.98 mg/g, depending on the treatment and species); however, these plants were at a different stage of development in comparison to plants tested in our experiment. In the mentioned study, when exposed to different salinity levels (4, 8, 12, 16 dS/m electrical conductivity levels of applied NaCl treatment solutions), plants with higher levels of proline showed only a slight change in proline content, while those with lower content reached similar final content with a greater increase in accumulation.

Restrepo et al. [19] studied the physiological response of three butternut squash cultivars (*C. moschata* Duchesne) to different irrigation regimes in two consecutive field trials located under a plastic roof without sidewalls. The content of MDA and proline significantly increased in plants exposed to drought. Proline and MDA accumulation were inversely proportional to water supply (1.69–2.14 mg/g fw and 530–670 nmol/g fw in more irrigated and 18.49–25.23 mg/g fw and 930–1250 nmol/g fw in the least irrigated plants, respectively). MDA levels were up to 14 times higher than in our study, which points to severe stress in *C. moschata* plants exposed to limited irrigation. The difference between the mentioned study and the results presented in this paper could be due to the transplantation of plants (which could cause additional stress in plants), different growing conditions (a field covered with a plastic roof without sidewalls), and different stages of development of sampled plants compared with our experiment.

Our study showed that when leaves from the F I treatment were compared among species, *C. moschata* plants had values similar to those of the other two species (Figure 1, Supplementary Figure S1, Supplementary Table S2). Conversely, the lack of irrigation increased MDA levels up to 50.8% in *C. moschata* plants. Additionally, there was no difference between the 0F+0I and F+0I treatments for all tested species, but differences were statistically significant when these treatments were compared with irrigated ones (Supplementary Table S2).

Sheikhnavaz Jahed et al. [20] determined the effect of priming (hydro-priming, gibberellin, GR24—a strigolactone, benzyl aminopurine and spermidine) on the germination of aged (control, 85% and 75% of control germination) pumpkin seeds (*Cucurbita pepo* L.) under different water potentials obtained by polyethylene glycol 6000 (0, −0.75 and −1.5 MPa). Their results showed that the lowest content of proline (0.55 μmol/g fw) was in the control seeds and the highest (1.57 μmol/g fw) was in the drought treatment with an intensity of −1.5 MPa of water potential, when 75% aged seeds were primed with spermidine. Also, the authors concluded that seed aging has a greater impact on the reduction in morphological and biochemical parameters than drought stress. Another study performed on *C. pepo* [16] showed the impact of Se-fortification on cellular integrity, leaf nutrient content, and osmotic adjustment of plants grown under salt stress in open field conditions. The MDA content of control plants was 500 nmol/g fw and up to 200 nmol/g fw in plants exposed to stress. A similar increasing trend was observed for proline and glutathione contents (up to 2.3-times increase in stress-exposed plants).

Tested *C. maxima* and *C. moschata* plants that were fertilized and/or irrigated had higher GSH contents than plants from the 0F+0I treatment (Figure 3). Both fertilization and irrigation had a positive impact on GSH accumulation (2.7–5.4 mg/g); however, plants that were only fertilized but not irrigated, in the majority of cases, had higher accumulation of this antioxidant compound (Figure 3), but the increase was not higher than 10.9%. In treatments where plants received water, fertilization had no impact on accumulation of GSH, which could point to the effect of irrigation on GSH levels in this experiment, rather than fertilization itself (Figure 3 and Supplementary Figure S1).

Reduced glutathione (γ-glutamylcysteinylglycine, GSH) plays many important roles in plant cells (reviewed in [27]). Reduced glutathione serves as a storage and transport molecule of sulfur, and it participates in the regeneration of antioxidant molecules and accumulation of anthocyanins, as well as in the protection of thiol groups of proteins under stress conditions. Additionally, GSH represents a precursor for phytochelatins, which are synthesized under cadmium or other toxic metal exposures. The ability of glutathione reductases to keep the GSH/GSSG (reduced/oxidized glutathione) redox balance under control has been shown to be important for the tolerance of the plant to drought stress, since GSH participates as a reducing agent in the ascorbate–glutathione pathway, which is important for ascorbate peroxidases (APx) recycling [28] and the reduction of α-tocopherol and zeaxanthin in cell membranes [27]. Studies that measured the level of GSH under extreme temperatures, salt and drought stress, especially those with external application of GSH, confirmed that GSH increases under stressful conditions and that external addition of GSH significantly reduces MDA content [27].

Total phenolics content (ranging from 8.6 to 38.4 mg/g GAE) was mostly higher in plants from the F+I treatment, especially for *C. pepo* genotypes (Figure 4c). Additionally, *C. maxima* genotypes had higher TP content in leaves of plants from the 0F+0I treatment. When compared among species, *C. moschata* genotypes generally had significantly lower total phenolic content in leaves than the other two species (Supplementary Figure S1 and Supplementary Table S2).

Phenolic compounds, as complementary antioxidants, are participants in crosstalk with ROS during acclimation of plants to various stresses (damage induced by physical agents, UV, high temperatures, drought, salinity, etc.). It is well-known that the content and composition of phenolics depend on the genotype, developmental stage and growing conditions and their accumulation is usually associated with stress mitigation [15,29]. Flores-Leon [13] demonstrated that chlorophyll content, relative water content, and flavonol accumulation (measured by a non-destructive device) constitute reliable physiological indicators of tolerance to drought and combined heat–drought stress, with direct relevance for breeding applications.

Correlation analyses among tested parameters within tested treatments showed that there is a significant negative relationship between LP and TP for 0F+0I (r = −0.68), F+0I (r = −0.68), and F+I (r = −0.77) treatments (Supplementary Table S3). This could highlight the accumulation of phenolic compounds as a feature of importance for mitigating the consequences of exposure to abiotic stress in tested plants. Contrary to these treatments, there was no significant correlation between LP and TP (r = −0.11) within the 0F+I treatment, which was probably a result of low variability in the results measured for LP and low intensity of LP in leaves of tested plants (Figure 1 and Supplementary Figure S1).

Figure 5 shows results of PCA for all measured data in R with *FactoMineR* and *factoextra* packages. PC1 had 39.29% of the variance, while PC2 had 28.33% variance, together accounting for 67.62% of the variance (Figure 5a). Proline and TP made the greatest contributions to PCs (Figure 5b) and were positively correlated with PC2 (r = 0.92) and PC1 (r = 0.77), respectively (Supplementary Table S4). Additionally, LP was in a negative correlation with PC1 (r = −0.80) and was in third place in the scree plot that represents the eigenvalues (as a percentage of variance) ordered from largest to smallest, following Pro in the first and TP in the second place (Figure 5a and Supplementary Table S4). Figure 5c shows a correlation plot of variables and their contributions (%), in which variables that point in the same direction are positively correlated. Additionally, the length of the arrows represents how well variables are represented.

As shown in Figure 6a, *C. moschata* samples were clearly separated from *C. maxima* and *C. pepo*. When tested parameters are compared among the species for plants receiving both fertilization and irrigation, significant differences can be observed. Most of the *C. moschata* samples had high LP and Pro contents under limited water supply conditions (0F+0I and F+0I), while *C. pepo* and *C. maxima* had higher contents of TP, GSH and Pro. If individual samples are compared, those positioned close together have more similar multivariate profiles and their position in the graph points to correlation with contributing parameters (Figure 5 and Figure 6). Samples that are in the second quadrant of PC analysis (between Pro and TP arrows) can be recommended as promising candidates for further testing in drought-prone conditions, due to high correlation with Pro and TP and negative correlation with LP, especially samples that performed well in the 0F+0I treatment (mostly *C. maxima* genotypes: Max12-1, Max37 and Max113).

Drought stress stimulates accumulation of phenolic compounds that have a protective role against oxidative stress by increasing plant scavenging capacity and enhancing drought stress tolerance. Higher levels of MDA in plants under drought stress indicate more oxidative damage and severity of stress faced by plants, due to the inability of antioxidants to scavenge ROS [30]. Screening different genotypes under suboptimal conditions may provide valuable information on the strategy that plants use to adapt to unfavorable growing conditions.

## 3. Materials and Methods

Genotypes tested in this research were selected for the experiment due to their morphological characteristics that were important for further breeding processes. These genotypes were partially pre-evaluated in previous multiyear field studies, in terms of both fruit nutritional quality and agronomic yield [31,32]. They represent a part of the *Cucurbita* species collection (circa 400 accessions) from the Institute of Field and Vegetable Crops, National Institute of the Republic of Serbia.

Nine genotypes of *C. maxima* and *C. moschata* and seven genotypes of *C. pepo* (Table 1) were grown in an open field experiment at the Institute of Field and Vegetable Crops (coordinates: 45°20′ N, 19°51′ E) during 2025.

The field is located on a calcareous chernozem on a loess terrace. The chemical soil properties were pH 8.12 (in H_2_O) and 7.21 (in KCl); 0.19% (total N); 1.85% CaCO_3_; 3.62% humus content and available P and K contents—31.14 and 31.10 mg/100 g soil, respectively. The sowing was performed on 25 April 2025.

The experiment (randomized complete block design) was organized in three blocks (repetitions). Each block had four treatments: (1) no fertilization and no irrigation (0F+0I), (2) no fertilization and irrigation (0F+I), (3) fertilization and no irrigation (F+0I), and (4) fertilization and irrigation applied (F+I). Seven plants per treatment in the block for each genotype were grown in rows (21 plants per treatment and genotype). Plants were spaced 1.5 m apart within rows, with 5.0 m between rows (Supplementary Figure S2).

The plants were grown following standard regional cultivation practices, including applying 16:16:16 NPK fertilizer at the rate of 200 kg/ha (in late November), mechanical and chemical weed control, and taking plant protection measures as required. The drip-irrigation system was active 6 h per day. The dripper type was compensating, with equal flow at different pressures (working pressure approx. 1.5 bar). Pipes had 1.1 mm wall thickness, a diameter of 16 mm, a dripper spacing of 33 cm, and a water flow of 2.3 L per hour on each dripper. The effective irrigation volume received by the plants was approx. 50 L per day. The dripper system provided uniform watering directly to the root zone of the plants and it maintained the same amount of water along the entire row.

The official reports of the Republic Hydrometeorological Service of Serbia (RHSS, 2025) [33] were used to describe the weather conditions during the pumpkin growing season for the area of Novi Sad (April–September 2025). Monthly values of agrometeorological parameters from April to September 2025 are given in Figure 7.

According to reports of RHSS, June 2025 was the driest in the last 55 years. The significant precipitation deficit combined with high air temperatures caused severe drought in the region. Based on the Z-index (Figure 7), extreme drought prevailed over a large part of the country in June (−5.5), while the rest of the season (July–September) had moderate to severe drought (−1.3 to −3). The same period had PDSI values from −2.8 to −3.9, which point to severe drought.

The Palmer Drought Severity Index (PDSI) is a long–term climatic water balance index, and it is regarded as a comprehensive representation of soil–water balance, since it uses readily available temperature and precipitation data to estimate relative dryness [34]. PDSI estimates soil moisture as the difference between precipitation and evapotranspiration. PDSI ranges from −1.9 to 1.9 and represents near-normal moisture conditions. Negative PDSI numbers represent long-term moisture deficits (−2 to −2.9 moderate; −3 to −3.9 severe; <−4 extreme drought), while positive numbers represent long-term moisture surpluses [33]. Z index, or Palmer’s Z index of Drought, represents a short-term climatic water balance index, since it is more responsive to short-term anomalies (rapidly developing drought). It differs from PDSI values, with a range from −1.24 to 0.99 that refers to near-normal moisture conditions. Negative values for Palmer’s Z index represent short-term moisture deficits (−1.25 to −1.0 moderate; −2 to −2.74 severe; <−2.75 extreme drought), while positive numbers represent short-term moisture surpluses [33].

Leaf samples (the third fully expanded leaves from the top of the vine) were collected in the final two decades of July 2025. All plants were at the reproductive phase of plant development after emergence of the first female flower (the beginning of the flowering stage) or 6.01. stage according to Phenological growth stages and BBCH-identification keys of cucurbits [35]. Although all samples were collected at the same stage, stress-induced flowering delays may have affected developmental comparability.

Third leaves are fully expanded leaves selected due to their role as a carbon source in contrast to young leaves, which act as carbon sinks before achieving full expansion [36]. Additionally, older leaves were avoided due to potential senescence and mechanical injuries. Furthermore, flower abortion most often occurs at nodes located further from the plant’s roots [10,11,37], so it was important to compare tested stress markers at the flowering stage to understand the level of oxidative stress in these leaves. All sampling was performed in the early morning due to lower temperatures and minimal insolation, to prevent additional stress to plants.

Four leaves were collected from each genotype and each treatment (three biological repetitions per treatment). All leaves were cut into disks using the cork borer (avoiding major leaf veins). Some of the sampled leaf disks were used for preparation of buffer extracts, and a part of the leaves were frozen (−25 °C) and then freeze-dried. Leaves from each biological replicate were extracted in triplicate for each of the applied methods (technical repetitions).

Buffer extracts were prepared using one g of leaves macerated in a chilled mortar with a pestle, adding 10 mL (2 × 5 mL) of phosphate buffer (0.1 M KH_2_PO_4_; pH = 7, pH adjusted with KOH). The homogenate was further centrifuged for 10 min at 4 °C and 5000 rpm and the supernatant was used for analyses of lipid peroxidation and reduced glutathione content.

Freeze-drying of frozen leaf disks was performed under vacuum, with 20 min of additional freezing at −80 °C and then 24 h of main drying under chamber pressure of 0.1 mbar at −80 °C (Alpha 2-4 LSC basic, Martin Christ, Germany). Freeze-dried leaves were used for analyses of total phenolics and proline contents (all extractions were performed three times).

All spectrophotometric measurements were performed using a PerkinElmer UV/Vis Lambda 25 spectrophotometer.

The end products of membrane lipid breakdown in cells, as a result of lipid peroxidation (LP), were measured as malondialdehyde (MDA) equivalents produced. The method for determination of lipid peroxidation intensity was explained in Hodges et al. [38] and Jambunathan [39]. The reaction medium contained buffer extract of fresh pumpkin leaves (0.5 mL) and a mixture (3 mL) of 0.5% thiobarbituric acid (TBA) and 20% trichloroacetic acid (TCA). After incubation of the reaction medium at 90 °C for 30 min, the change in color was recorded at 532 and 600 nm (a nonspecific absorbance A_600_ was subtracted from A_532_). The concentration of MDA was calculated using the Beer–Lambert equation (the extinction coefficient of MDA is 155 mM^−1^ cm^−1^). The total amount of TBARS (TBA-reactive substances) was expressed as nmol malondialdehyde (MDA) equivalents/g fresh weight (nmol MDA/g fw).

Total proline content was determined by the method described by Bates et al. [40]. The reaction medium contained 0.7 mL each of 0.2% ninhydrin, glacial acetic acid, and supernatant of plant extract obtained by 10 min centrifugation at 5000 rpm of 25 mg freeze-dried leaves and 2 mL 3% sulfosalicylic acid aqueous solution. After 1 h of boiling the reaction mixture in a water bath at 100 °C, the reaction was stopped by cooling the tubes in an ice bath for 5 min. The formed chromophore was mixed with 2 mL of toluene by vigorous shaking, after which the absorbance of the upper layer was measured at 520 nm. The concentration of proline was estimated by referring to a standard curve (y = 0.0806x + 0.0997; r^2^ = 0.98; 0–35 µg/mL) for L-proline and expressed as mg proline equivalents per g of dry weight (mg Pro/g dw).

Reduced glutathione content (GSH) was determined by reaction of Ellman’s reagent, 5,5-dithiobis-(2-nitrobenzoic acid) (DTNB), with non-protein thiol (-SH) and measurement of color changes at 412 nm, according to Sedlak and Lindsay [41]. One mL each of the buffer extract, 5% trichloroacetic acid (TCA), and distilled water was mixed and centrifuged for 10 min at 5000 rpm at 4 °C. Supernatant (0.1 mL) was added to a medium consisting of 2.0 mL 0.4 M TRIS-HCl buffer (pH 8.9), 0.9 mL H_2_O and 0.1 mL 6 mM DTNB. The content of GSH in the sample was calculated from the standard curve (y = 0.0448x; r^2^ = 0.99; 0–23 µg/mL) of dependence of absorbance on GSH concentration and expressed as the number of mg GSH equivalents per gram of fresh weight (mg GSH/g fw).

The content of total phenolics in 50% ethanol extracts was determined by the Folin–Ciocalteu reagent (slightly modified method explained by Makkar et al. [42]). Dried leaf powder (1 g) was extracted with 10 mL of 50% ethanol in a cooled ultrasonic bath for 1 h. The extracts were then centrifuged for 10 min at 6000 rpm. Each supernatant was filtered through the Chromafil AO-45/25 polyamide filter produced by Macherey–Nagel (Düren, Germany). The reaction medium contained 5 mL of distilled water, ethanolic extract (200 μL), and Folin’s reagent (0.5 mL, water: Folin = 2:1, *v*/*v*). After 5 min, 1 mL of 20% Na_2_CO_3_ was added to the reaction medium and left for an hour to incubate at room temperature and in a dark place. The absorbance of the reaction medium was measured at 756 nm against the blank containing distilled water instead of the extract. A dilution series of gallic acid was used to construct the calibration curve (y = 0.0935x + 0.0156, r^2^ = 0.99; 0–12 µg/mL). The content of total phenolics is expressed as mg of Gallic Acid Equivalents per g of dry weight (mg GAE/g dw).

To account for the randomized block design, a mixed-model Multivariate Analysis of Variance (MANOVA) was performed to evaluate the effects of Genotype, Treatment and Species, and their combinations on tested biochemical parameters. The model utilized a sigma-restricted parameterization approach to accommodate categorical predictors. The multivariate significance of the main effects and interactions was evaluated using Wilks’ lambda. No adjustments for missing data were required. Residuals were evaluated for normality (using the Shapiro–Wilk’s W test); no data transformations were necessary, and all analyses were performed on the raw data. Genotype or Species (depending on the set of data), Treatment, and their interaction were specified as fixed effects, whereas Blocks were included as a random effect. Significant multivariate main effects and interactions were followed by a two-way Factorial ANOVA (Genotype × Treatment). Post hoc comparison of the means was performed using Tukey’s Honestly Significant Difference (HSD) test, with statistical significance set at *p* < 0.05. These data were analyzed using Statistica 12 (StatSoft, Tulsa, OK, USA). Pearson correlation analysis within applied treatments was performed in the same program.

Principal component analysis (PCA) was performed to evaluate relationships among measured parameters across pumpkin genotypes and treatments using the R environment (R Core Team, 2026, R version 4.6.1 [43]). Data handling, statistical procedures and visualization were performed using the *caret*, *corrplot*, *devtools*, *factoextra, FactoMineR*, *ggbiplot*, *ggfortify*, *ggplot2*, *patchwork*, and *tidyverse* packages [44,45,46,47,48,49,50,51,52,53]. Adjustment of fonts and conversion into appropriate formats are performed using *showtext*, *magick*, and *pdftools* packages [54,55,56].

## 4. Conclusions

The results presented in this paper were obtained from a field trial conducted during a single growing season and should therefore be regarded as preliminary. To assess the potential of the examined parameters as indicators for breeding pumpkins for drought tolerance, their variation should be evaluated in relation to agronomic characteristics. The trial will therefore be continued over multiple growing seasons, with the expected inter-seasonal variation in drought intensity providing an opportunity to better elucidate the relationships between the investigated biochemical parameters (at different developmental stages) and agronomic performance of the tested pumpkins.

Our results showed that there are differences among tested *Cucurbita* species, as well as among their genotypes, when it comes to tested biochemical markers and their response to contrasting irrigation and fertilization conditions. These findings represent a valuable tool in selecting potentially promising candidate genotypes for further drought-tolerance evaluation.

The use of climate-resilient cultivars in cases of limited water supply and fertilizer availability provides food production stability and can help mitigate the impacts of climate change on the cultivation of tested species.

## Figures and Tables

**Figure 1:**
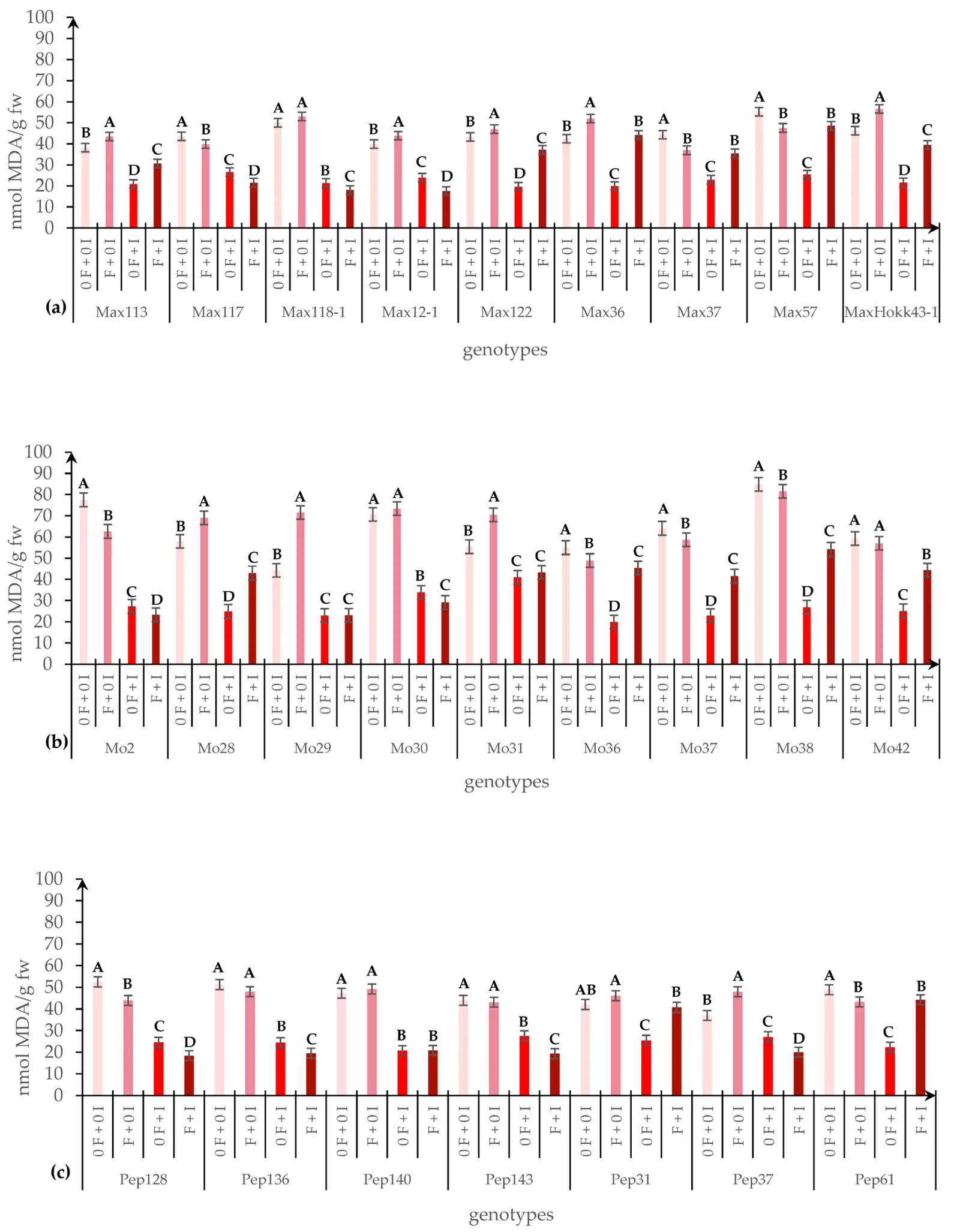
Lipid peroxidation intensity in leaves of *C. maxima* (**a**), *C. moschata* (**b**) and *C. pepo* (**c**) genotypes from different treatments: no fertilization and no irrigation (0F+0I), fertilization and no irrigation (F+0I), no fertilization and irrigation (0F+I), and fertilization and irrigation (F+I) applied. Results within the genotypes marked with different letters differ significantly at *p* < 0.05 (Tukey’s HSD test). Values of tested biochemical parameters were expressed as means ± standard error determined for three biological replicates.

**Figure 2:**
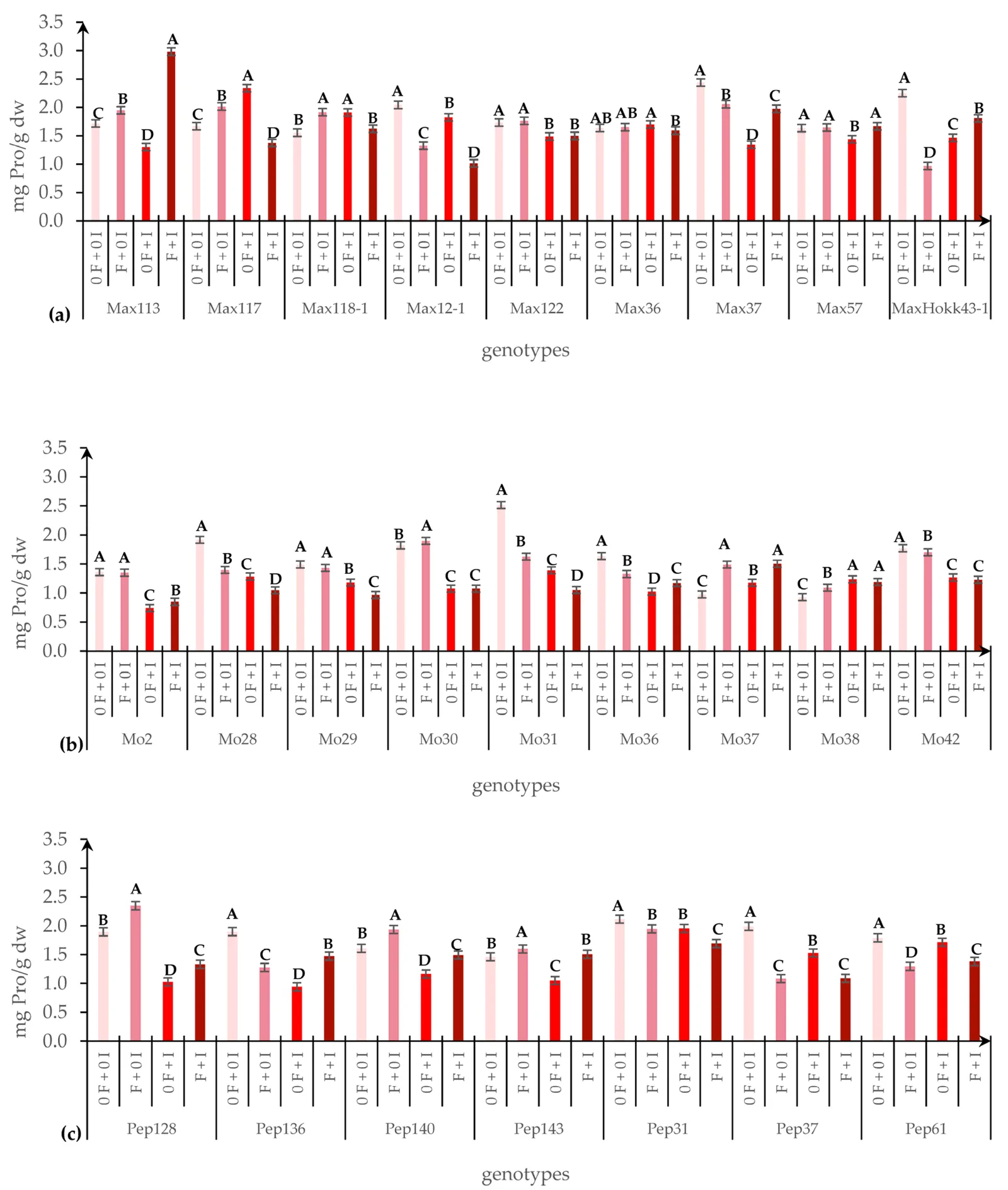
Proline content in leaves of *C. maxima* (**a**), *C. moschata* (**b**) and *C. pepo* (**c**) genotypes from different treatments: no fertilization and no irrigation (0F+0I), fertilization and no irrigation (F+0I), no fertilization and irrigation (0F+I), and fertilization and irrigation (F+I) applied. Results within the genotypes marked with different letters differ significantly at *p* < 0.05 (Tukey’s HSD test). Values of tested biochemical parameters were expressed as means ± standard error determined for three biological replicates.

**Figure 3:**
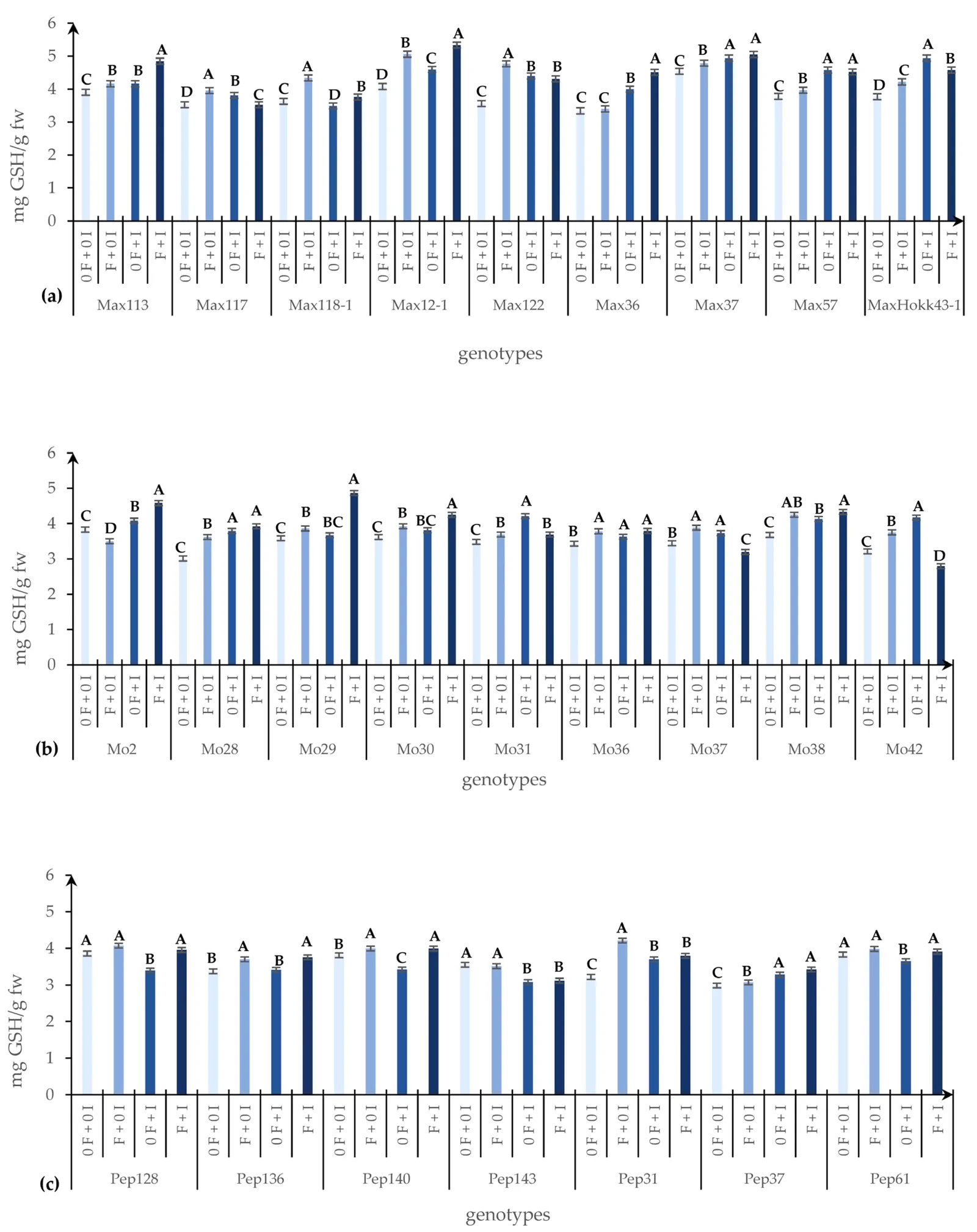
Reduced glutathione content in leaves of *C. maxima* (**a**), *C. moschata* (**b**) and *C. pepo* (**c**) genotypes from different treatments: no fertilization and no irrigation (0F+0I), fertilization and no irrigation (F+0I), no fertilization and irrigation (0F+I), and fertilization and irrigation (F+I) applied. Results within the genotypes marked with different letters differ significantly at *p* < 0.05 (Tukey’s HSD test). Values of tested biochemical parameters were expressed as means ± standard error determined for three biological replicates.

**Figure 4:**
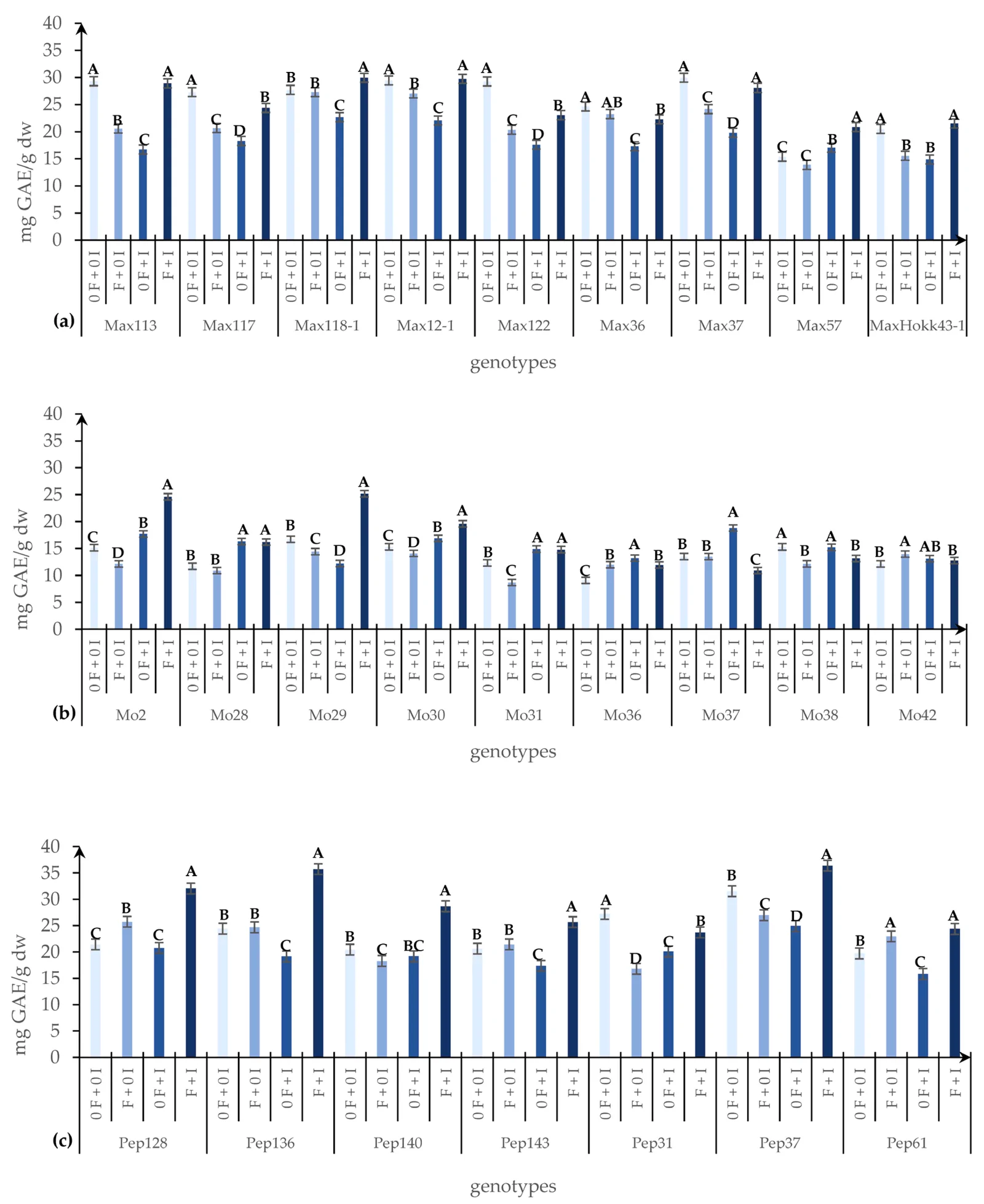
Total phenolic content in leaves of *C. maxima* (**a**), *C. moschata* (**b**) and *C. pepo* (**c**) genotypes from different treatments: no fertilization and no irrigation (0F+0I), fertilization and no irrigation (F+0I), no fertilization and irrigation (0F+I), and fertilization and irrigation (F+I) applied. Results within the genotypes marked with different letters differ significantly at *p* < 0.05 (Tukey’s HSD test). Values of tested biochemical parameters were expressed as means ± standard error determined for three biological replicates.

**Figure 5:**
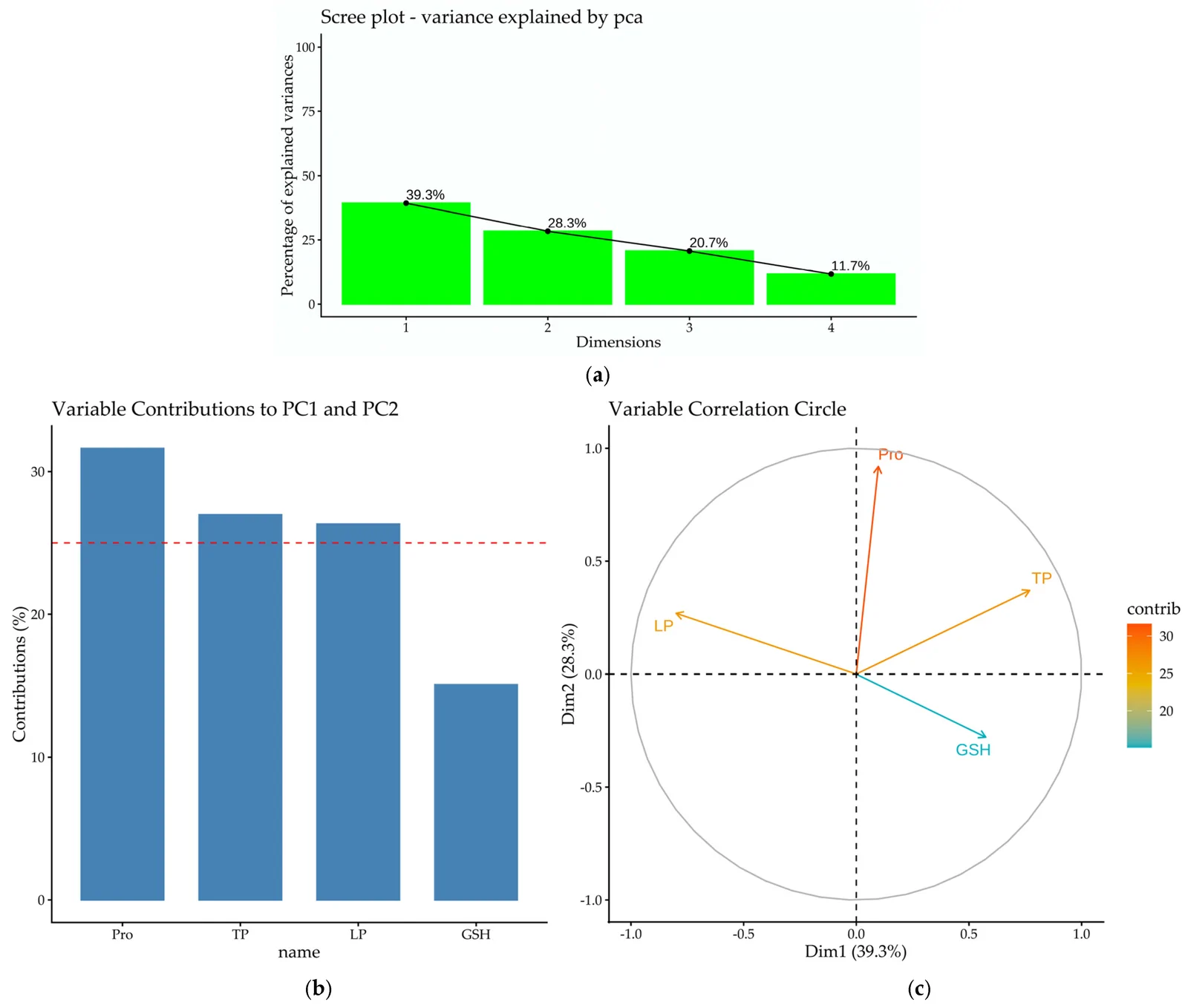
Multivariate integration of tested parameters (LP—lipid peroxidation intensity, Pro—proline content, GSH—reduced glutathione content, TP—total phenolics) in leaves of *C. maxima*, *C. moschata* and *C. pepo* genotypes from different treatments: (**a**) scree plot: the eigenvalues (as a percentage of variance) ordered from largest to smallest; (**b**) variable contributions to PC1 and PC2, the red dashed line is the expected average contribution; (**c**) variable correlation circle; the color scale represents the contribution of the parameter (from lower—teal, to higher orange), while arrows represent the correlation among parameters.

**Figure 6:**
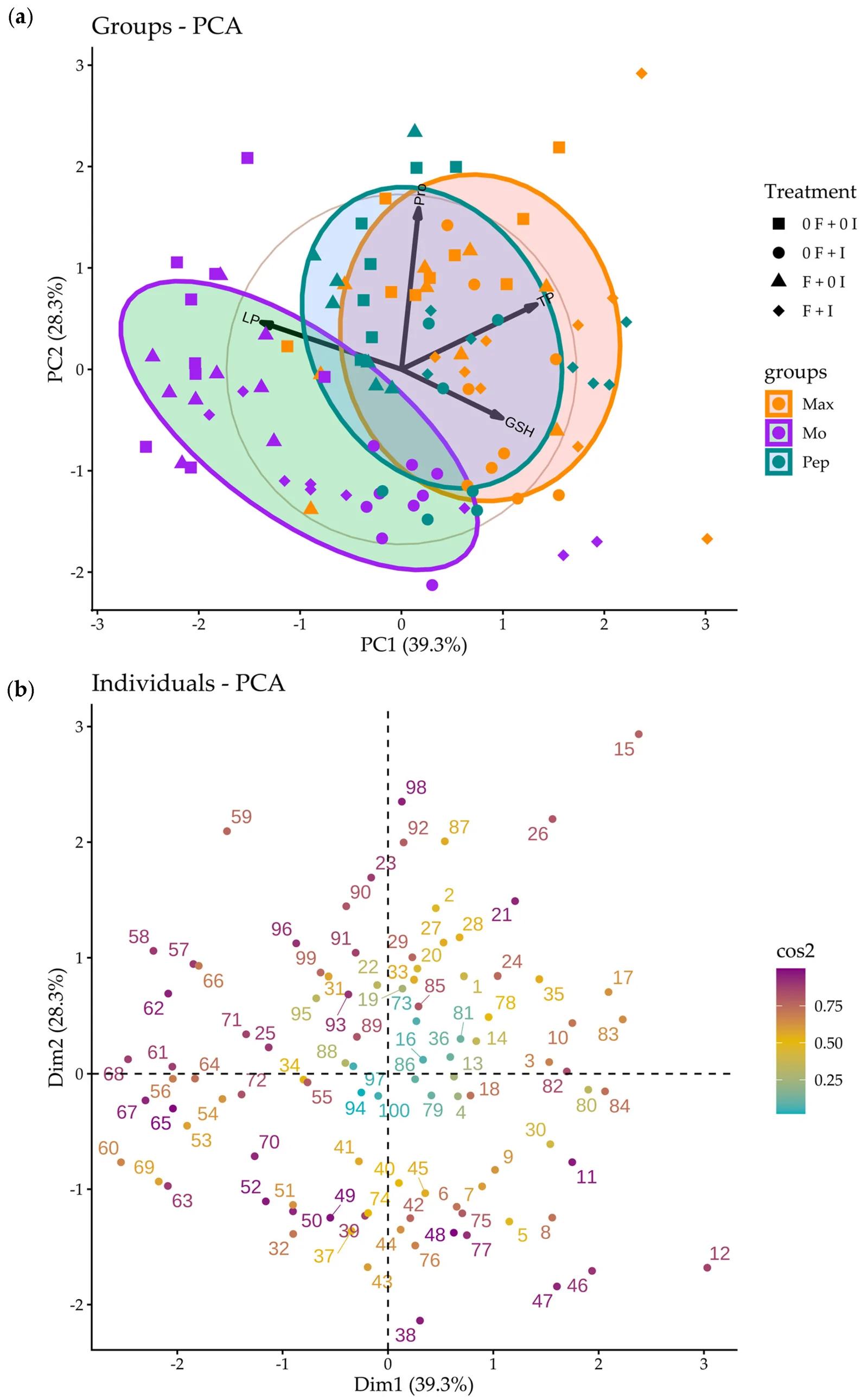
Principal component analysis (PCA) of tested parameters (LP—lipid peroxidation intensity, Pro—proline content, GSH—reduced glutathione content, TP—total phenolics) in leaves of *C. maxima*, *C. moschata* and *C. pepo* genotypes: (**a**) groups—PCA performed by species and different treatments: no fertilization and no irrigation (0F+0I), fertilization and no irrigation (F+0I), no fertilization and irrigation (0F+I), and fertilization and irrigation (F+I) applied. Arrows represent the correlations among parameters and samples; (**b**) individuals—PCA analysis performed by individual samples (Supplementary Table S5). High cos2 means the individual is well represented on these two dimensions (PCs).

**Figure 7:**
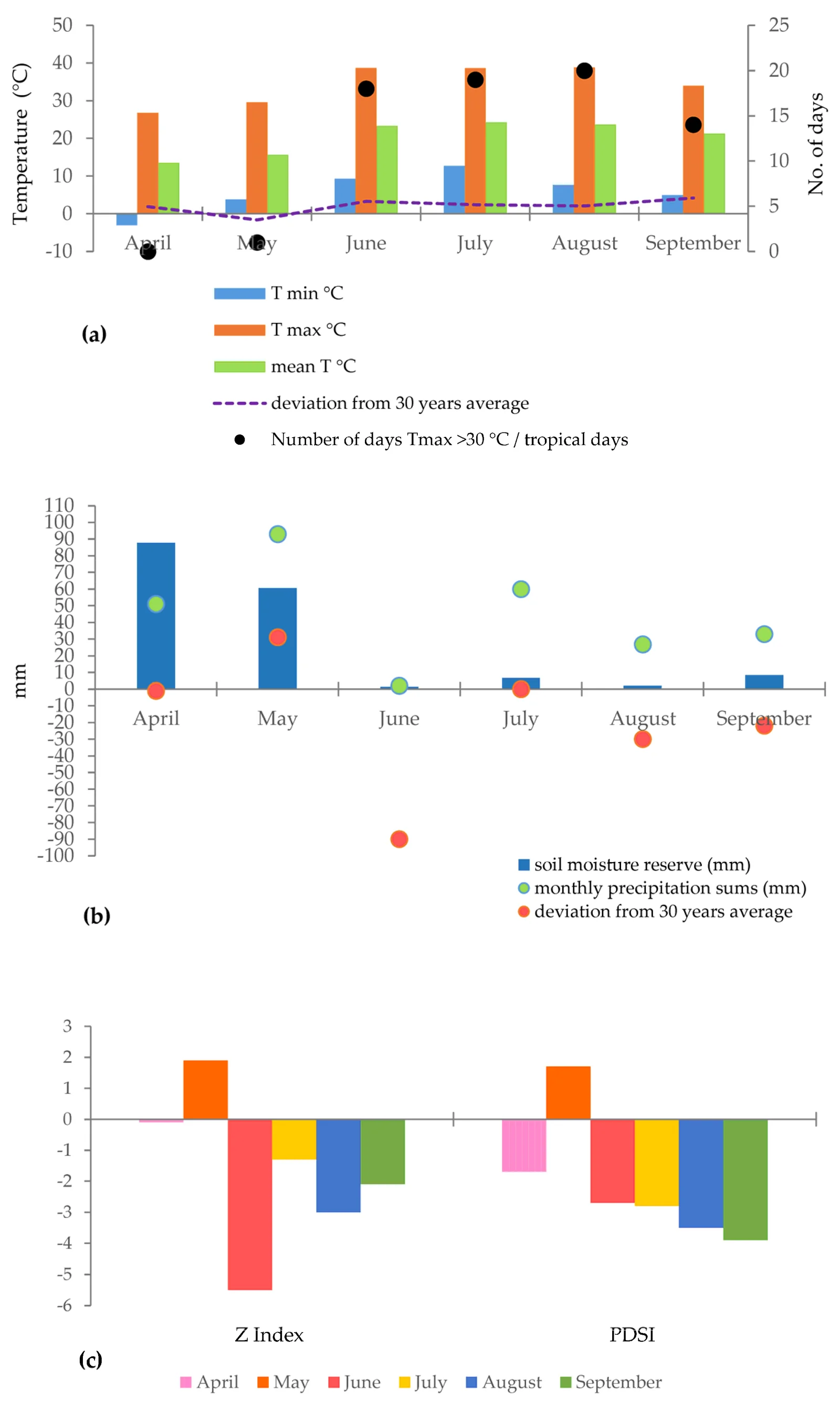
Monthly values of agrometeorological parameters from April to September 2025: (**a**) Temperature, (**b**) Precipitation, (**c**) Z index—Palmer’s Z index of Drought/Moisture and PDSI—Palmer Drought Severity Index.

**Table 1:** *C. maxima*, *C. moschata* and *C. pepo* genotypes from the *Cucurbita* species IFVCNS collection.

*C. maxima* Genotypes	*C. moschata* Genotypes	*C. pepo* Genotypes
Sample Name	Sample Name	Sample Name
Max113	Mo2	Pep128
Max117	Mo28	Pep136
Max118-1	Mo29	Pep140
Max12-1	Mo30	Pep143
Max122	Mo31	Pep31
Max36	Mo36	Pep37
Max37	Mo37	Pep61
Max57	Mo38	
MaxHokk43-1	Mo42	

## Data Availability

The data supporting the findings of this study are available from the authors upon request.

## Supplementary Materials

The following supporting information can be downloaded at https://www.mdpi.com/article/10.3390/plants15192971/s1: Table S1. Multivariate Analysis of Variance (MANOVA) of the effects of Genotype, Species, Treatment and their combinations on tested biochemical parameters. *C. maxima* (Max), *C. moschata* (Mo) and *C. pepo* (Pep) genotypes from tested treatments: no fertilization and no irrigation (0F+0I), fertilization and no irrigation (F+0I), no fertilization and irrigation (0F+I), and fertilization and irrigation (F+I); Table S2. Effect of Species, Treatment and Species × Treatment on lipid peroxidation intensity (LP), total phenolics (TP), proline (Pro) and reduced glutathione (GSH) contents in leaves of *C. maxima* (Max), *C. moschata* (Mo) and *C. pepo* (Pep) genotypes from different treatments: no fertilization and no irrigation (0F+0I), fertilization and no irrigation (F+0I), no fertilization and irrigation (0F+I), and fertilization and irrigation (F+I). nd—no statistical difference, *—there is a significant difference between the species at *p* < 0.05 (Tukey’s HSD test); Table S3. Pearson correlation matrix showing relationships among tested parameters (LP—lipid peroxidation intensity, TP—total phenolics, Pro—proline content, GSH—reduced glutathione content) in leaves of *C. maxima*, *C. moschata* and *C. pepo* accessions from different treatments: no fertilization and no irrigation (0F+0I), fertilization and no irrigation (F+0I), no fertilization and irrigation (0F+I), and fertilization and irrigation (F+I). Correlations marked in red (font color) are significant at *p* < 0.05. Colors in the table cells (highlights) represent correlation coefficients ranging from negative (dark green) to positive (white) associations; Table S4. Principal Component Analysis (PCA) in R with FactoMineR and factoextra packages; Table S5. A list of all genotypes and treatments represented by a number in Figure 6b; Figure S1. Box plot of tested biochemical markers in leaves of *C. maxima*, *C. moschata* and *C. pepo* genotypes from different treatments: no fertilization and no irrigation (0F+0I), fertilization and no irrigation (F+0I), no fertilization and irrigation (0F+I), and fertilization and irrigation (F+I). Distribution of individual values within treatments is represented by dots; Figure S2. A scheme of the experimental design.

## Abbreviations

The following abbreviations are used in this manuscript:

LP	Lipid peroxidation
TP	Total phenolics content
Pro	Proline content
GSH	Reduced glutathione content
